# Revolutionizing the Pancreatic Tumor Diagnosis: Emerging Trends in Imaging Technologies: A Systematic Review

**DOI:** 10.3390/medicina60050695

**Published:** 2024-04-24

**Authors:** Sabina Florina Șolea, Mihaela Cristina Brisc, Alexandra Orășeanu, Florian Ciprian Venter, Ciprian Mihai Brisc, Răzvan Mihai Șolea, Lavinia Davidescu, Amina Venter, Ciprian Brisc

**Affiliations:** 1 Doctoral School of Biological and Biomedical Sciences, University of Oradea, 410087 Oradea, Romania; sabi_florina95@yahoo.com (S.F.Ș.); adaoraseanu@yahoo.com (A.O.); cypry_85@yahoo.com (F.C.V.); razvanm1989@yahoo.com (R.M.Ș.); aminaadnan2005@yahoo.com (A.V.); brisciprian@gmail.com (C.B.); 2 Bihor Clinical County Emergency Hospital, 410169 Oradea, Romania; 3 Department of Medical Disciplines, Faculty of Medicine and Pharmacy, University of Oradea, 410073 Oradea, Romania; 4 Faculty of Medicine and Pharmacy, University of Oradea, 410068 Oradea, Romania; brisciprian08@gmail.com (C.M.B.); lavinia.davidescu@yahoo.com (L.D.)

**Keywords:** elastography, gastroenterology, pancreatic tumors, pancreatitis, imaging, ultrasound

## Abstract

*Background and Objectives*: The pancreas, ensconced within the abdominal cavity, requires a plethora of sophisticated imaging modalities for its comprehensive evaluation, with ultrasonography serving as a primary investigative technique. A myriad of pancreatic pathologies, encompassing pancreatic neoplasia and a spectrum of inflammatory diseases, are detectable through these imaging strategies. Nevertheless, the intricate anatomical confluence and the pancreas’s deep-seated topography render the visualization and accurate diagnosis of its pathologies a formidable endeavor. The objective of our paper is to review the best diagnostic imagistic tools for the pancreas. *Materials and Methods*: we have gathered several articles using Prisma guidelines to determine the best imagistic methods. The imperative of pancreatic scanning transcends its diagnostic utility, proving to be a pivotal element in a multitude of clinical specialties, notably surgical oncology. Within this domain, multidetector computed tomography (MDCT) of the pancreas holds the distinction of being the paramount imaging modality, endorsed for its unrivaled capacity to delineate the staging and progression of pancreatic carcinoma. In synergy with MDCT, there has been a notable advent of avant-garde imaging techniques in recent years. These advanced methodologies, including ultrasonography, endoscopic ultrasonography, contrast-enhanced ultrasonography, and magnetic resonance imaging (MRI) conjoined with magnetic resonance cholangiopancreatography (MRCP), have broadened the horizon of tumor characterization, offering unparalleled depth and precision in oncological assessment. Other emerging diagnostic techniques, such as elastography, also hold a lot of potential and promise for the future of pancreatic imaging. Fine needle aspiration (FNA) is a quick, minimally invasive procedure to evaluate lumps using a thin needle to extract tissue for analysis. It is less invasive than surgical biopsies and usually performed as an outpatient with quick recovery. Its accuracy depends on sample quality, and the risks include minimal bleeding or discomfort. Results, guiding further treatment, are typically available within a week. Elastography is a non-invasive medical imaging technique that maps the elastic properties and stiffness of soft tissue. This method, often used in conjunction with ultrasound or MRI, helps differentiate between hard and soft areas in tissue, providing valuable diagnostic information. It is particularly useful for assessing liver fibrosis, thyroid nodules, breast lumps, and musculoskeletal conditions. The technique is painless and involves applying gentle pressure to the area being examined. The resulting images show tissue stiffness, indicating potential abnormalities. Elastography is advantageous for its ability to detect diseases in early stages and monitor treatment effectiveness. The procedure is quick, safe, and requires no special preparation, with results typically available immediately. *Results*: The assembled and gathered data shows the efficacy of various techniques in discerning the nature and extent of neoplastic lesions within the pancreas. *Conclusions*: The most common imaging modalities currently used in diagnosing pancreatic neoplasms are multidetector computed tomography (MDCT), endoscopic ultrasound (EUS), and magnetic resonance imaging (MRI), alongside new technologies, such as elastography.

## 1. Introduction of Comprehensive Diagnostic and Management Strategies for Pancreatic Cysts and Neoplasms

The diagnostic evaluation of a pancreatic mass is a systematic process that integrates clinical assessment, laboratory testing, and advanced imaging techniques to determine the nature of the lesion.

Initially, a comprehensive patient history and physical examination are conducted to identify symptoms and risk factors associated with pancreatic diseases. Laboratory tests follow, including blood work to check liver function and tumor markers such as CA 19-9 and CEA, which may suggest malignancy if elevated. Imaging studies play a pivotal role; abdominal ultrasonography often serves as the first-line imaging modality due to its accessibility and non-invasiveness. This is typically followed by more definitive imaging with computed tomography (CT) or magnetic resonance imaging (MRI), which provide detailed views of the pancreatic anatomy and the surrounding structures that are crucial for assessing the mass’s characteristics and its impact on adjacent tissues. Endoscopic ultrasound (EUS) with fine needle aspiration (FNA) is frequently employed for both imaging and biopsy, allowing for cytological or histopathological examination of the mass. This step is critical for distinguishing between benign and malignant lesions and determining the appropriate management strategy. Collectively, these diagnostic steps enable the comprehensive evaluation of pancreatic masses, facilitating targeted therapeutic interventions based on the specific pathology identified.

For example, pancreatic cysts are fluid-filled sacs that form in the pancreas, a vital organ in the digestive system responsible for producing enzymes that aid in digestion and the production of hormones such as insulin that regulate glycemia. These cysts vary widely in their potential to cause symptoms or lead to more serious conditions, including pancreatic cancer. The incidence of pancreatic cysts tends to increase with age, and they are often discovered incidentally during imaging tests for other conditions [1].

There are several types of pancreatic cysts, each with unique characteristics and associated risks. The most common types include intraductal papillary mucinous neoplasms (IPMNs), mucinous cystic neoplasms (MCNs), serous cystadenomas (SCAs), and pseudocysts. IPMNs are particularly notable for their potential to become cancerous, especially those located in the main pancreatic duct. MCNs, more common in younger women, also carry a risk of malignancy but are typically found in the body and tail of the pancreas. SCAs are generally benign but can cause discomfort as they grow. Pseudocysts are often related to pancreatitis and are less likely to be precancerous [2].

The management of pancreatic cysts is predicated on a multifaceted approach that considers the cyst’s type, size, symptomatic presentation, and the patient’s general health status. This strategy is essential for the effective oversight of these cysts and for mitigating potential risks associated with their progression. Regular surveillance and comprehensive medical assessments are imperative to distinguish between benign and malignant characteristics of the cysts. Benign cysts often require less aggressive management and are monitored for changes in size or onset of symptoms, whereas malignant cysts necessitate a more proactive approach due to the high risk of cancer progression. Diagnosing these traits accurately involves detailed imaging studies and may also require cytological evaluation through fine needle aspiration to assess the nature of the cyst. Therefore, a tailored management plan that incorporates regular monitoring and dynamic medical evaluation is crucial to address the specific needs dictated by the benign or malignant properties of pancreatic cysts [2].

A research study [3] assessed the efficacy of various imaging techniques—CT, MRI, and EUS—in diagnosing pancreatic neoplasms, particularly pancreatic adenocarcinoma, among a cohort of 140 patients, of whom 100 were diagnosed with pancreatic cancer and 40 with other pancreatic masses. The study provided critical insights into the localization of pancreatic tumors, predominantly found in the head of the pancreas of 64-year-old urban males. In terms of diagnostic accuracy, CT achieved 83.3%, MRI 89.1%, and EUS 82%. However, the precision of EUS significantly improved to 93.7% when supplemented by contrast-enhancement and elastography techniques, especially for tumors smaller than 20 mm, where EUS was notably superior. Most diagnoses occurred at advanced stages (III and IV), underscoring the need for early detection. Both CT and MRI were instrumental in identifying metastases in liver, peritoneal, and extra-abdominal sites. The findings underscore the critical importance of accurately diagnosing both malignant and benign pancreatic formations. Enhanced EUS techniques were particularly effective for initial tumor identification, while CT and MRI were crucial in mapping metastatic spread, emphasizing the need for a multimodal imaging approach to optimize the management and therapeutic strategies for patients with pancreatic tumors.

When trying to diagnose pancreatic cystic lesions, especially when using enhanced ultrasound for the quantification of tumor perfusion, the clinician should always consult the current guidelines [4].

It is noteworthy that some investigations [5] indicate a tendency to overutilize imaging for acute pancreatitis diagnosis, even when abdominal discomfort and elevated serum amylase or lipase levels already confirm the condition. This overuse of computed tomography (CT) scans not only increases healthcare costs but also does not improve the management or shorten hospital stays for patients with uncomplicated acute pancreatitis. These investigations can also help us to understand how to better diagnose cystic pathologies of the pancreas, which are difficult to diagnose due to the pancreas’s anatomy and location.

## 2. Material and Methods

To create this article, an extensive literature review was carried out using several databases, encompassing PubMed, Scopus, and Web of Science. The bibliography was carefully selected based on the pertinence and relevance of the articles to pancreatic imaging, emphasizing the diagnostic precision and efficiency of multiple imaging modalities such as CT, MRI, and EUS, among plenty of others. Every article was carefully assessed, and various insights were gathered and studied to present an exhaustive perspective on the prevailing trends in pancreatic imaging. The assortment of articles covered plenty of research areas, from comparative examinations and the assessment of particular imaging methods to explorations into the excessive application of some techniques. The insights gleaned from these scholarly sources constitute the foundation of the discourse in this article.

Having undertaken this exploration across multiple databases from PubMed, Scopus, and Web of Science, we selected a collection of 100 articles pertinent and relevant to pancreatic imaging that aligned with the criteria set for this review piece. Through this retrospective examination, our goal is to have a collective understanding regarding the best imaging technique for diagnosing both malignant and benign pancreatic tumors.

To ensure the relevance and quality of our review, we established certain criteria for article inclusion. Initially, from the 100 articles we selected after consulting the inclusion and exclusion criteria, we eliminated 22 articles due to duplication across the databases. Following this, we conducted a thorough review of the remaining 78 articles. During this process, we excluded 14 articles as they did not meet our predefined criteria.

**Figure 1:**
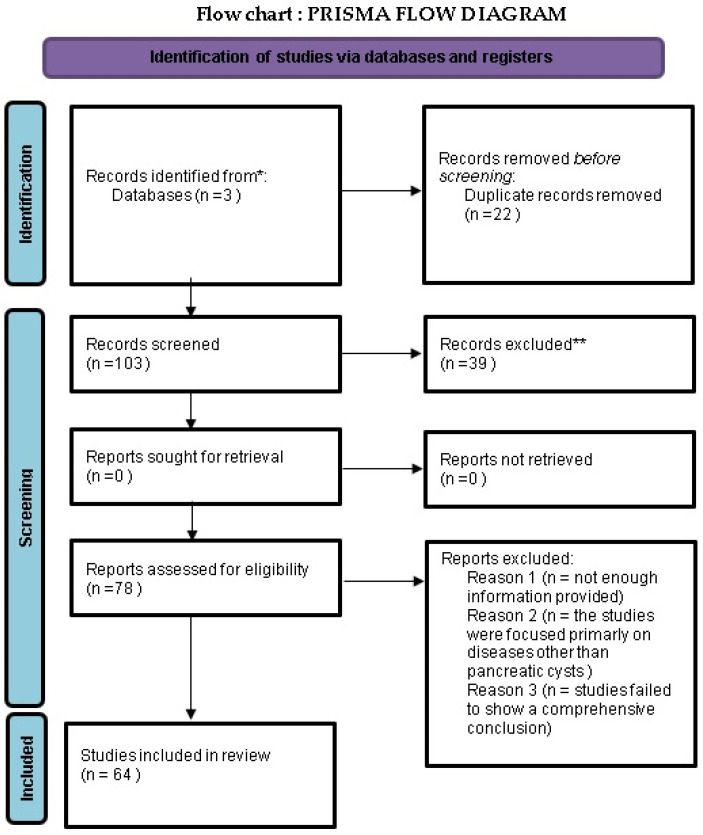
Prisma Flow Diagram explaining selection of articles. * Database consisted of PubMed, Scopus, and Web of Science. ** Records were excluded from article according to our exclusion criterias.

As a result of our screening and evaluation process, we identified 64 articles that met our high standards for inclusion in this comprehensive review. These articles are central to our discussion and in-depth analysis of the current advancements in pancreatic imaging techniques. The selection and exclusion of articles were methodically documented in a PRISMA flow diagram (Figure 1), as shown below in the flow chart with the same name (Flow Chart: Prisma Flow Diagram). This diagram is not just a visual representation but also a testament to the thoroughness and transparency of our research methodology.

The rigorous nature of our selection process ensures that this review is founded on research that is not only relevant but also of the highest quality. Such a foundation is crucial for providing an authoritative and dependable overview of state-of-the-art pancreatic imaging. We delved into each article, examining the methodologies, results, and implications of the studies. This allowed us to not only summarize the current knowledge in the field but also to identify gaps and propose directions for future research.

Moreover, our review does not just present the findings; it contextualizes them within the broader landscape of medical imaging. We discuss the implications of these advanced techniques for clinical practice, highlighting how they can improve diagnosis, treatment planning, and patient outcomes. We also consider the challenges and limitations of current technologies, offering a balanced view that acknowledges both the strides made and the hurdles yet to be overcome. In order to accomplish this, we have established some rigorous inclusion and exclusion criteria.

Inclusion Criteria:

1. Patients Diagnosed with Pancreatic Tumors: Individuals confirmed to have pancreatic tumors through preliminary imaging or biopsy.
2. Availability of Imaging Data: Patients must have undergone one or more of the specified imaging techniques (ultrasonography, MDCT, MRI, EUS, CEUS, or elastography) prior to the study.
3. Age: Adults aged 18 years and older.
4. Consent to Participate: Patients willing to provide consent for their imaging data to be reviewed and included in the study.

Exclusion Criteria:

1. Lack of Confirmed Diagnosis: Individuals without a confirmed diagnosis of a pancreatic tumor.
2. Incomplete Imaging Data: Patients without complete imaging studies relevant to the research.
3. Underage Individuals: Patients under the age of 18.
4. Non-consenting Individuals: Patients unwilling or unable to provide consent for their data to be used in the study.

In conclusion, this review serves as an essential resource for clinicians, researchers, and students alike, offering a detailed and nuanced understanding of the latest developments in pancreatic imaging. It is our hope that this work will not only inform but also inspire further research and innovation in this vital field of medicine.

This extended passage builds upon our original text by adding depth and context, emphasizing the thoroughness of the research process, the implications of the findings, and the broader impact on the field of medical imaging.

## 3. Commonly Used Imagistic Methods for Diagnosis

**Figure 2:**
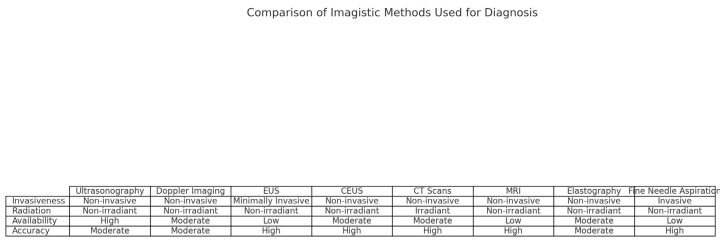
The comparison of different imagistic methods for pancreatic lesions.

When considering imagistic methods for diagnosis, clinicians usually try to opt for the most painless, noninvasive, and non-radiant methods. Thus, the most commonly used method is ultrasonography, which is quick and readily available and can be done as many times as needed in order to determine a diagnosis [3,4]. Doppler imaging is another method in which the clinician can determine the blood flow surrounding the organ, which can help to determine the characteristics of the formation. Other classical methods include EUS, CEUS, CT scans, and MRI. Elastography is another noninvasive method in which one can map the characteristics of the tumor, such as the stage of fibrosis, which can lead to a diagnosis; it is an emerging technique that shows a lot of promise for the future. Other methods can be used, such as fine needle aspiration; however, this is a much more invasive method. On the other hand, fine needle aspiration gives a more accurate description of the characteristics of the tumoral formation (malign or benign). These main findings are explained more concisely in Figure 2.

Diagnosing pancreatic pathologies and distinguishing between benign and malignant lesions is a complex task that presents several challenges, even with the use of advanced imaging techniques such as ultrasonography, Doppler imaging, endoscopic ultrasound (EUS), and magnetic resonance imaging (MRI). These modalities, each with their unique properties and limitations, are critical tools in the clinical decision-making process.

Ultrasonography is a noninvasive, widely available technique that provides real-time images and is invaluable for initial assessments. However, its utility is often limited by its lower resolution and the difficulty of penetrating obese patients, which can obscure the detailed features necessary for differentiating between types of pancreatic lesions. Consequently, while it serves as a primary tool for identifying anomalies, its diagnostic specificity for pancreatic conditions is often inadequate without supplementary imaging techniques.

Doppler imaging enhances ultrasonography by assessing the blood flow characteristics around pancreatic lesions, offering clues about their nature based on vascular patterns. Increased vascularity can suggest malignant potential, but such indications are not definitive and can overlap with inflammatory processes.

EUS stands out for its higher resolution imaging capabilities closer to the pancreas, facilitated by the proximity of the transducer to the target tissues via the gastrointestinal tract. This proximity allows EUS to not only visualize the pancreatic architecture in detail but also to perform guided fine needle aspirations. These biopsies can provide tissue samples for cytological evaluation, which is crucial for confirming malignancy. However, EUS is minimally invasive and its effectiveness is highly dependent on the operator’s expertise, which can vary significantly.

MRI provides excellent soft tissue contrast and can be enhanced with magnetic resonance cholangiopancreatography (MRCP) to provide noninvasive ductal imaging. It is particularly useful in delineating cystic from solid lesions and identifying subtle structural changes. However, the high cost, limited availability, and longer examination times restrict its routine use in some clinical settings.

Despite these advanced technologies, the overlapping imaging features of benign and malignant pancreatic lesions frequently lead to diagnostic ambiguities. Malignant lesions may present as well-defined masses similar to benign cysts, and inflammatory processes can mimic the appearance of cancerous growths. The interpretation of these images requires a high degree of clinical expertise and often necessitates a multidisciplinary approach involving radiologists, pathologists, and gastroenterologists to achieve a definitive diagnosis.

## 4. Benign and Malign Pancreatic Tumors

Pancreatic tumors are classified into benign and malignant types, each characterized by distinct biological behaviors, prognostic implications, and clinical management approaches.

Benign pancreatic tumors are non-cancerous growths that do not invade the surrounding tissue nor metastasize to distant parts of the body. Examples include serous cystadenomas and mature teratomas.

These tumors generally have a favorable prognosis and may not necessitate aggressive therapeutic interventions.

Conversely, malignant pancreatic tumors, such as pancreatic adenocarcinoma—which originates from the epithelial cells lining the pancreatic ducts—are characterized by their potential to invade adjacent structures and metastasize, which significantly worsens prognosis [3,4].

### 4.1. Diagnosis of Pancreatic Tumors

The diagnostic process for benign pancreatic tumors begins with a comprehensive patient history and physical examination to document symptoms and any familial predisposition to cancers. This is followed by blood tests, which might include markers such as CA 19-9, although these are not specific to benign conditions. Initial imaging often involves an ultrasound, which is followed by computed tomography (CT) or magnetic resonance imaging (MRI) to delineate the tumor’s size and location more clearly. A biopsy is rarely necessary unless there is ambiguity regarding the tumor’s nature [2].

In diagnosing malignant pancreatic tumors, similar initial steps are taken. The patient’s history and physical examination are crucial for noting any indicative signs such as jaundice, unexplained weight loss, or abdominal pain. Blood tests are expanded to include tumor markers such as CA 19-9 and CEA to support a diagnosis of malignancy. Imaging studies play a critical role, with ultrasound usually serving as the preliminary assessment tool. CT and multidetector CT scans are instrumental for staging the cancer, evaluating its resectability, and planning potential surgical intervention. MRI, combined with magnetic resonance cholangiopancreatography (MRCP), provides comprehensive imaging of the pancreatic and biliary duct systems. Endoscopic ultrasound (EUS) is particularly valuable for its dual role in detailed imaging and facilitating fine needle aspiration (FNA) for cytological analysis. In some cases, laparoscopy may be employed to verify the extent of disease spread and to validate staging, further informing the treatment strategy.

The rigor of this diagnostic process reflects the complexity and severity of pancreatic tumor pathologies, underscoring the necessity for precise and thorough medical evaluation.

### 4.2. Imaging Steps for Diagnosis of Pancreatic Tumors

After taking the necessary blood tests, the clinician must decide on the best imagistic methods to use. Usually, the first imagistic tool used is the conventional ultrasound, due to its advantages of being noninvasive, painless, and without any radiation exposure. Depending on the nature of the tumor depicted on the ultrasound, the examiner can choose from CEUS, CT, EUS, MRI, and, as of late, elastography to help determine the characteristics of the tumor.

### 4.3. Imagistic Methods: Ultrasonography and It’s Multiple Uses

Transabdominal ultrasound (US) is widely recognized as the initial imaging method for diagnosing pancreatic lesions.

However, to distinguish between inflammatory and malignant lesions, as well as between pseudocysts and cystic tumors, contrast techniques are essential. Contrast-enhanced (CE) ultrasonography has emerged as a valuable tool in this context, offering a performance comparable to that of contrast-enhanced computer tomography/magnetic resonance imaging (CT/MRI). Its advantages include being safer and non-irradiant; however, a special preparation might be needed in patients with renal pathologies and high levels of creatinine if contrast substance is used [4]. As per the current guidelines on the use of contrast-enhanced ultrasound (CEUS) [4], this technique is particularly beneficial for enhancing the characterization of ductal adenocarcinoma; differentiating pseudocysts from cystic tumors; distinguishing vascular (solid) from avascular (liquid/necrotic) components of a lesion; more accurately defining the dimensions and margins of a lesion, including its relationship with adjacent vessels; and aiding in selecting subsequent imaging techniques.

The pancreas, being a retroperitoneal organ, poses challenges for evaluation via ultrasound due to the poor acoustic window created by intestinal gas. Therefore, an experienced operator is crucial for accurate assessment. While ultrasonography is effective in identifying pancreatic lesions, it falls short in differential diagnosis and staging, particularly in cases of suspected malignant tumors. Contrast enhanced ultrasonography (CEUS), although not useful for detecting focal pancreatic lesions, is instrumental in characterizing ultrasound-detected lesions and solid tumors, such as ductal adenocarcinoma or neuroendocrine tumors, and in differentiating pseudocysts from pancreatic cystic tumors [1].

Conventional ultrasound followed by contrast-enhanced ultrasonography can swiftly assess the pancreatic lesion’s pattern and characterize its vascularization, enabling an immediate differential diagnosis post-detection. CEUS stands out as a real-time imaging method that allows for continuous visualization of vascular enhancement patterns, unlike the snapshot approach of CT or MRI. Its lack of side effects and irradiation also means CEUS can be repeated immediately if results are inconclusive without further damaging the patient’s health.

In addition to the above, ultrasonography has a multitude of advantages, namely that it is a cheap, non-invasive, quick, and mostly accurate way of diagnosing a patient. Masses are typical seen on the pancreas using ultrasonography; however, the sensitivity and accuracy of the diagnosis largely depends on a number of factors, such as the experience of the operator or the stadialization of the disease. Because of this, ultrasonography is seen as a slightly controversial method of diagnosing with accuracy, having an accuracy of anywhere between 50% and 90% [6,7,8].

Ultrasound can also be a helpful complimentary tool to diagnosis using other methods. This is seen in endoscopic ultrasound (EUS), which is a much more sensitive way of diagnosing a patient.

Some diseases, such as Groove pancreatitis [9] (also known as paraduodenal pancreatitis or cystic duodenal dystrophy), which is a rare form of pancreatic cystic pathology and a focal form of chronic pancreatitis seen in alcoholics, is localized in between the duodenum, pancreatic head, and common biliary duct. Due to the way in which the possible fibrosis in Groove pancreatitis spreads, it can often be confused with carcinoma. By using EUS [10], the operator can view the histological modifications that appear as a result of changes in pancreatic viscosity due to nicotine abuse or alcohol excess. These histological changes include pancreatic duct calcification, enzyme flow impairment, Brunner gland hyperplasia, and duodenal wall cysts [11].

Noninvasive methods are preferred when working with the pancreas due to its position in the body and its general sensitivity to trauma or severe illness. Disconnected pancreatic duct syndrome is a severe necrotic pancreatitis with pancreatic duct disruption with a prevalence of 10–31% [12]. In such severe cases, the best imaging method for diagnosis is a combination of noninvasive MRI and MRCP due to their capabilities to show both the pancreas and peripancreatic changes and to analyze the proximal and distal ends of the ruptured main pancreatic duct and possible fistulas [13].

### 4.4. Imagistical Metshods: Doppler Ultrasound Evaluation

Doppler ultrasound is a noninvasive medical test that uses reflected sound waves to evaluate blood as it flows through a blood vessel. It helps doctors assess blood flow through major arteries and veins, such as those of the arms, legs, and neck [7]. It can reveal blood clots, blocked or narrowed blood vessels, tumors in vessels, and congenital vascular malformations. There are different types of Doppler ultrasound, including Color Doppler, which adds color to the image to show the speed and direction of blood flow. Doppler ultrasound is a valuable tool for diagnosing various medical conditions related to blood flow. In the context of pancreatic diagnostics, Doppler ultrasound is adept at examining pancreatic cysts by assessing the vascular characteristics within the cyst walls or septa. This capability is instrumental in distinguishing between benign and malignant pancreatic cysts. Typically, malignant cysts exhibit increased blood flow, a phenomenon associated with tumor angiogenesis. This increased vascularity is a critical marker detectable via Doppler ultrasound, aiding the differentiation of cystic lesions. However, this method is supplementary and usually part of a more comprehensive diagnostic approach, including other imaging techniques such as CT scans or MRI. Doppler ultrasound helps provide a more complete understanding of the nature of pancreatic cysts [7].

### 4.5. Imagistical Methods: Elastography in the Diagnosis of Pancreatic Tumors

Elastography has emerged as a transformative imaging technique since its inception in the early 1990s, particularly for diagnosing pancreatic diseases and conditions involving superficial organs such as the breast or thyroid. Initially applied to these more accessible organs, elastography has expanded to include the deeper, parenchymatous organs of the digestive system. Despite its capabilities, the retroperitoneal location of the pancreas presents challenges for transabdominal ultrasound elastography, leading to the adoption of more precise methods such as endoscopic ultrasound (EUS) elastography. This technique, often combined with fine-needle aspiration (EUS-FNA), significantly enhances the diagnostic accuracy for pancreatic conditions by allowing detailed assessments of tissue stiffness. Advances in elastography include the development of shear wave elastography, which provides more accurate measurements of tissue stiffness, and the integration of artificial intelligence to improve reliability and reduce errors [14]. Research has shown that pancreatic tissue stiffness varies with age, illustrating the diagnostic versatility of elastography [15].

Elastography also enables the measurement of strain index values (SI) across different pancreatic regions, assessing correlations with demographic factors and aiding in the early detection of pathological changes in children and adults alike [16,17]. Despite its potential, further research is necessary to refine elastography’s applications and validate its clinical efficacy. Quantitative shear wave elastography (SWE) has been particularly useful in distinguishing between benign and malignant pancreatic masses, with studies confirming its high diagnostic accuracy through elevated shear wave velocities in malignant cases [18]. This method’s potential for routine clinical use promises to reduce unnecessary procedures and enhance the cost-effectiveness of pancreatic tumoral disease management, although its broader adoption requires further standardization and validation in diverse clinical settings.

### 4.6. Imagistical Methods: EUS Used in Benign and Malignant Pancreatic Cystic Diseases

Since its inception in the 1980s, endoscopic ultrasonography (EUS) [19] has undergone significant evolution, transitioning from a purely diagnostic tool to a pivotal instrument in therapeutic interventions and minimally invasive surgeries. This evolution has expanded its utility in addressing a broad spectrum of both benign and malignant medical conditions [10], particularly those affecting the gastrointestinal tract, mediastinum, and critical organs such as the pancreas, liver, and kidneys. It works by defining the status of fibrosis of an organ in real time as the examiner assesses the pancreatic tissue strain index.

In recent times, EUS has increasingly been applied to more complex, invasive procedures traditionally reserved for percutaneous or surgical approaches. This expansion is largely due to the continual advancement of techniques and the development of sophisticated devices, which have enhanced both the diagnostic and therapeutic capabilities of EUS. Despite these advancements, the field of endoscopic ultrasonography faces ongoing challenges, including the need for formalized training programs and the establishment of comprehensive guidelines to standardize the use of these innovative techniques.

Technological progress in EUS has significantly enhanced its diagnostic precision. Cutting-edge techniques such as EUS-guided elastography and contrast-enhanced EUS have become integral, especially in managing pancreatic pathologies. These methods are crucial for differentiating benign from malign formations and distinguishing between different types of pancreatic tumors, thereby underscoring the essential role of EUS in the contemporary clinical landscape [14]. EUS’s refined imaging capabilities enable the detailed visualization that is necessary to differentiate benign from malignant pancreatic tumors, providing a critical tool in the diagnostic arsenal for oncological assessments.

### 4.7. CT Scans and Their Usefulness and Flexibility in Pancreatic Pathologies

In the realm of computed tomography (CT) imaging, particularly when contrast agents are employed, the preprocedural evaluation of renal function emerges as a pivotal consideration. This practice is primarily rooted in the mitigation of contrast-induced nephropathy (CIN), a notable risk particularly in patients with pre-existing renal insufficiencies [4]. The kidneys play a central role in filtering and excreting contrast materials; hence, compromised renal function can lead to suboptimal clearance of these agents, potentially culminating in an accumulation that poses a significant health hazard. Consequently, the assessment of renal function is instrumental in tailoring the dosage of contrast media to individual patient needs, thereby aligning with the principles of personalized medicine. This approach is particularly salient in identifying patients at heightened risk of renal complications, such as those with chronic kidney disease, diabetes, or hypertension, and older people. Establishing a baseline renal function is also crucial, as it facilitates a comparative analysis in the event of postprocedural renal impairment, thereby aiding the attribution of causality to the contrast agent, if necessary. Moreover, this preemptive evaluation guides clinical decision-making, balancing the diagnostic benefits of contrast-enhanced CT against the potential renal risks and, in certain scenarios, prompting the consideration of alternative diagnostic modalities. Additionally, in cases where renal compromise is detected, it enables the planning of prophylactic measures, such as hydration therapy or post-scan monitoring, to support renal function and mitigate associated risks. In essence, the pre-scan assessment of renal function is not merely a procedural formality but a critical component of patient-centric care in the context of contrast-enhanced CT imaging, underscoring its significance in contemporary radiological practices [3,5,13].

Previous research has aimed to assess the diagnostic accuracy of transabdominal ultrasound and computed tomography (CT) in detecting chronic pancreatitis, a condition marked by repeated inflammation of the pancreas leading to progressive damage, including the appearance of pancreatic cysts. The study compared the effectiveness of CT and ultrasound (US) against the modified Mayo score—a comprehensive standard that includes a variety of test results and clinical symptoms. Traditionally, CT scans are favored for diagnosing this condition, but advancements in ultrasound technology have positioned it as a viable contender. The inquiry involved examining 73 patients under suspicion of chronic pancreatitis through both CT and ultrasound, including endoscopic ultrasound. These imaging results were then classified according to standard criteria. The outcome revealed that CT and ultrasound share comparable efficiency in diagnosing chronic pancreatitis, with both methods achieving sensitivities of around 66% (correctly identifying approximately 66% of true chronic pancreatitis cases) and specificities of around 80% (accurately identifying about 80% of cases without the condition) [20].

When analyzing the prevalence of unwarranted CT scans among acute pancreatitis patients, it was discovered that, of 405 patients, 210 (51.85%) received CT scans without exhibiting severe symptoms of the disease. Only one patient (0.47%) showed evidence of complications from acute pancreatitis. These unnecessary scans led to an average cost of $4510 per scan, amounting to a total expenditure of $947,056. Interestingly, this financial outlay did not influence the median hospital stay duration, which remained at 3 days both for patients who underwent scans and those who did not. The findings indicate a tendency towards the excessive use of CT imaging for patients with uncomplicated acute pancreatitis, contributing to avoidable healthcare costs and radiation exposure. Based on these observations, it is advisable to forgo CT imaging when acute pancreatitis can be diagnosed clinically and confirmed through biochemical tests. Minimizing these scans could reduce healthcare expenses and lower patients’ exposure to radiation. There is a clear need for further education among healthcare providers to limit unnecessary imaging, thereby cutting costs and enhancing patient care [21].

However, in situations where the multidetector computed tomography is not available, the clinician can choose the computed tomography method (CT). CT is currently the number one method of imaging used for the diagnosis of chronic pancreatitis. Chronic pancreatitis is a difficult pathology to diagnose in the early phases of the pathology due to many patients either having recurring inflammatory episodes as the dominant symptom or experiencing non-specific symptoms. These symptoms appear before functional or structural pancreatic changes are detectable. The current guidelines [22,23,24,25,26] always recommend a multimodal setup that includes multiple methods of imagistic diagnosis. In this way, several diagnostic criteria have been developed [27,28].

### 4.8. ERCP—Endoscopic Retrograde Cholangiopancreatography

Endoscopic retrograde cholangiopancreatography (ERCP) represents an advanced endoscopic technique that integrates endoscopy with fluoroscopic imaging for the diagnostic and therapeutic management of biliary and pancreatic ductal pathologies. This multifaceted procedure plays a pivotal role in addressing a spectrum of hepatobiliary and pancreatic conditions. ERCP’s utility extends beyond diagnostics, encompassing therapeutic interventions such as biopsy, brush cytology, and the remediation of ductal obstructions or leaks. Its execution demands profound proficiency in endoscopic and fluoroscopic methodologies, necessitating the involvement of an endoscopist with specialized training in gastroenterology.

Despite its considerable value in the clinical landscape of pancreaticobiliary disease management, ERCP is associated with inherent risks, including pancreatitis, infections, perforations, and hemorrhage. These potential complications underscore the importance of meticulous patient selection and procedural preparation. In the hands of seasoned practitioners, however, ERCP stands as an indispensable modality in the comprehensive care of complex hepatobiliary and pancreatic disorders, balancing its inherent risks against its significant therapeutic and diagnostic benefits [29].

### 4.9. Multidetector Computed Tomography and Endoscopic Ultrasound in the Diagnosis of Pancreatic Masses

Pancreatic masses are present in certain pathologies, such as benign and malign tumors, and they can be seen by using conventional ultrasonography such as echography. However, other imagistic methods, such as multidetector computed tomography (MCDT), have appeared recently that give a much higher-quality image and that have a better accuracy for diagnosis and future treatment references [30]. MCDT can create 3-D multiplanar reconstruction images with improved spatial and temporal resolution. This imaging method also helps with the accuracy of determining tumor masses and their involvement in the common bile duct, peripancreatic vascular anatomy, and pancreatic duct.

A second method of evaluation that has surpassed classical ultrasonography is endoscopic ultrasound. This method is much more sensitive than even multidetector computed tomography when it comes to the visualization of pancreatic tumors that are smaller in size than 3 cm [31]. These small tumors usually also allow for concurrent biopsy [32]. However, the quality of this method depends largely on the operator who is performing the examination [33,34].

It was found that in 88% of cases where there were findings of solid lesions inside the pancreatic tissue, diagnosis using MDCT is much more consistent with the final tissue diagnosis. Using MDCT, the correct diagnosis percentage was 62% for cystic lesions (benign) and 100% for solid-cystic. Thus, multidetector computed tomography has proven its higher value in the accurate diagnosis of the nature of the tissue contained in the pancreas [35,36].

## 5. Comparing Imaging Techniques for Pancreatic Masses and Pathologies Leading to Pancreatic Cysts as a Complication

Nowadays, we can evaluate the type of pancreatic cysts (PLCs) present in pancreatic tissue [37]. PLCs can be classified as true cysts, pseudocysts, or cystic neoplasms. True cysts and pseudocysts are considered benign, while cystic neoplasms may turn malignant. They are further categorized into serous and mucinous cystic neoplasms, intraductal papillary mucinous neoplasms, and solid pseudopapillary neoplasms. The differentiation of these types is crucial due to their varying malignant potential.

**Figure 3:**
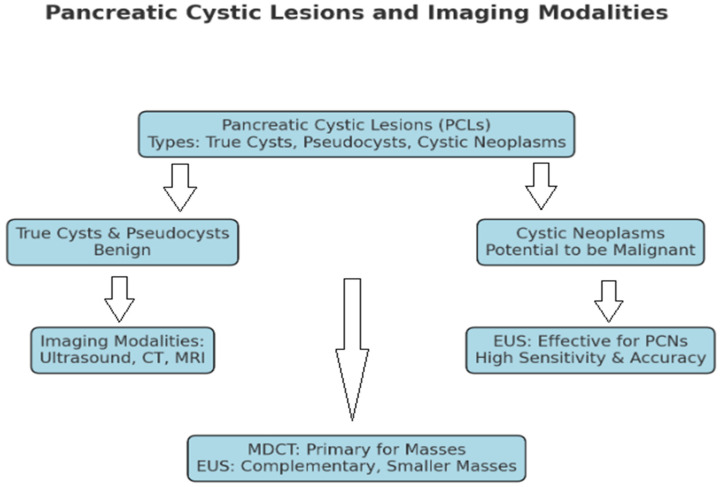
Pancreatic cystic lesions and imaging modalities.

An exploration of the diagnostic capabilities of ultrasound, CT, and MRI for identifying pancreatic cystic neoplasms (PCNs) reveals the superior accuracy of endoscopic ultrasound (EUS). EUS excels in differentiating non-neoplastic cysts from PCNs and provides a more effective characterization of PCNs than CT or MRI are able to. It demonstrates high sensitivity and accuracy in diagnosis, particularly in identifying key features, such as daughter cysts, septum, and papillae/nodules, which are essential for the precise classification of PCNs. The efficacy of EUS, however, hinges on the skill of the operator and carries a higher risk due to its invasive nature. The results are shown in Figure 3 [38].

Another issue arises when examining the diagnostic accuracy of CT. In comparing the accuracy of the diagnostic 256 multislice, CT, and endoscopic ultrasound (EUS) in assessing pancreatic masses, several conclusions were drawn [39]. A total of 36 patients with pancreatic masses were studied using these techniques and fine needle aspiration cytology (FNAC) when possible. Multidetector computed tomography (MDCT) and EUS were compared against histopathological findings. Overall, 83% of MDCT and 61% of EUS diagnoses were consistent with tissue diagnosis; however, the combined use of MDCT and EUS gave the best results. MDCT had a lower rate of inconclusive results than EUS. For determining the resectability of pancreatic adenocarcinoma, MDCT had 100% specificity and positive predictive value compared to 75% specificity and 92. 3% positive predictive value for EUS. The study concluded that MDCT is the most effective imaging method for the detection and characterization of pancreatic masses and for determining the resectability of malignant tumors. EUS is most beneficial for detecting smaller masses and for guiding FNAC. Overall, MDCT and EUS with EUS-guided FNA are complementary tools in the preoperative imaging of pancreatic masses. The two methods are not competitive but, rather, enhance each other’s strengths and make up for their limitations.

When [3] comparing CT, MRI, and EUS for diagnosing pancreatic adenocarcinoma, the study suggested that EUS, particularly when combined with contrast-enhancement and elastography, provided the highest diagnostic accuracy, but that CT and MRI remain crucial for detecting metastases. As a comparison of the specificity and sensibility of elastography as an enhancement tool using contrast enhancement and quantitative EUS, we used another study [40] on pancreatic masses [Table 1 and Table 2].

In terms of chronic pancreatitis, CT scans and ultrasounds were found to have similar diagnostic effectiveness, though neither method alone could definitively confirm a diagnosis. The overuse of CT scans in diagnosing acute pancreatitis was also highlighted, showing the need for the more judicious use of imaging in this context to prevent unnecessary costs and patient exposure to radiation.

Contrast-enhanced ultrasound (CEUS) has significantly advanced the field of medical imaging, offering an enhanced visualization of vascular structures and organ perfusion using gas-filled microbubbles as contrast agents. This technique has revolutionized diagnostic capabilities in various medical specialties, notably in oncology for differentiating benign from malignant lesions, providing critical insights into tumor vascularity that are essential for accurate cancer diagnosis [41]. In cardiology, CEUS has become a pivotal tool for noninvasively assessing myocardial perfusion and detecting myocardial infarction areas, offering an alternative to more invasive diagnostic methods [42].

The quantitative analysis of contrast enhancement patterns in CEUS is a rapidly expanding area of research, providing objective measurements for disease diagnosis and monitoring [43]. Beyond diagnostics, the potential of CEUS in therapeutic applications, such as targeted drug delivery and gene therapy, has been explored, leveraging the unique properties of microbubbles [44]. Technological advancements in CEUS, including the development of new contrast agents and the integration of AI for enhanced image analysis, are at the forefront of current research efforts [45].

Pediatric applications of CEUS, particularly due to its non-ionizing nature, have garnered significant interest, presenting a safer imaging alternative for children [46]. The role of CEUS in global health, especially in low-resource settings, is an area of active research, focusing on its cost-effectiveness and potential to improve healthcare accessibility [47]. Collectively, these developments in CEUS research and applications underscore its vital role in enhancing diagnostic and therapeutic processes in contemporary medical practice.

## 6. Pancreatic Biopsy—Fine Needle Aspiration

Fine needle aspiration (FNA), particularly when utilized in conjunction with endoscopic ultrasound (EUS), represents a cornerstone diagnostic tool for the assessment of pancreatic lesions, including both cystic and solid masses [31,34]. This technique, often referred to as fine needle biopsy (FNB) when solid tissue is involved, involves the use of a fine needle to extract cellular material for cytological examination. The utility of EUS-FNA in evaluating pancreatic cystic lesions smaller than 3 cm has been well documented, although it is occasionally omitted in cases lacking worrisome features or due to challenging lesion locations. Notably, when FNA is performed, it has demonstrated a high safety profile with no significant adverse events reported [48].

The diagnostic accuracy of EUS-FNA is further enhanced by analyzing carcinoembryonic antigen (CEA) levels and cytology results from the pancreatic cyst fluid (PCF). However, overlapping CEA values between malignant and non-malignant cysts suggest that CEA alone may not suffice as a definitive diagnostic marker [48]. Comparative studies of needle types, including standard versus flexible needles, have shown variations in success rates. Notably, flexible needles excel in accessing hard-to-reach regions such as the pancreatic head or the uncinate process [49]. The high correlation between cyst aspirate cytology and histopathology over a two-year follow-up underscores the method’s reliability, with only a minority of cases resulting in related serious adverse events.

Beyond cystic lesions, EUS-FNA is invaluable in the diagnosis of solid pancreatic masses, providing critical histopathological insights that guide clinical decision-making. The integration of molecular techniques, such as next-generation sequencing (NGS), has revolutionized the diagnostic landscape by identifying genetic mutations characteristic of specific pancreatic pathologies, thereby distinguishing between benign and malignant entities with greater precision [50]. Furthermore, the implementation of macroscopic on-site evaluation (MOSE) during FNA procedures allows for the immediate assessment of sample adequacy by a pathologist, enhancing diagnostic efficacy and potentially reducing the need for repeat biopsies.

By combining sophisticated imaging techniques, detailed cytological analysis, and advanced molecular diagnostics, clinicians are equipped to more accurately stratify patient risk and tailor management strategies effectively, be it through vigilant surveillance, surgical intervention, or alternative treatments. These advancements not only deepen our understanding of the nature and progression of pancreatic masses but also pave the way for more individualized and effective therapeutic approaches. This comprehensive approach highlights the indispensable role of FNA, extended into FNB for solid masses, in the modern diagnostic algorithm for pancreatic diseases.

## 7. Discussion on Assessing the Impact and Efficacy of Imaging Modalities in Pancreatic Cancer Diagnosis

In oncology, the precise diagnosis and treatment of pancreatic cancer are crucial due to its subtle symptoms and rapid advancement. Imaging plays a vital role in early detection and accurate staging. Pancreatic protocol CT offers detailed views for initial assessments, while MRI and MRCP excel in soft tissue contrast, highlighting vascular and ductal details. EUS combines endoscopy with ultrasound for biopsy and close-up imaging, enhancing diagnostic accuracy. PET scans, though less common initially, are key in spotting metastases and evaluating treatment efficacy [4].

European Society for Medical Oncology (ESMO) guidelines outline the imagistic diagnostic approach, positioning CT as the primary modality for detailing tumor characteristics and spread. MRI serves as an alternative when CT is inconclusive, with specific sequences for thorough analysis. EUS is reserved for staging specific cases and biopsies. The guidelines advocate for standardized reporting to ensure the uniform documentation of pancreatic cancer staging, emphasizing the limited role of ERCP compared to CT or MRI in diagnosis [51]. Recent studies underscore elastography’s diagnostic precision in identifying pancreatic tumors, highlighting its sensitivity and specificity in differentiating between malignant and benign lesions through endoscopic ultrasonography. This method, particularly strain elastography and shear wave elastography, has shown remarkable accuracy, reinforcing its value in medical diagnostics [50,51].

MRI’s role in diagnosing pancreatic tumors is expanding, offering detailed imaging without radiation exposure. Conventional EUS examination provides visual cues for diagnosing pancreatic cancer, including tumor appearance and vascular characteristics, while EUS-FNA boasts high sensitivity and specificity. EUS stands out for loco-regional staging, with enhancements such as contrast and elastography further improving its diagnostic capability [14]. Advancements in real-time tissue elastography (RTE) have introduced both qualitative and quantitative diagnoses, although variability in strain ratio measurements underscores the need for standardization. EUS’s ability to provide clear images and minimize interference makes it a preferred method for RTE in pancreatic evaluation [52,53,54,55,56].

## 8. Elastography: Exploring Recent Key Developments in Pancreatic Imaging Techniques

Elastography, a medical imaging technique that measures tissue elasticity, has significantly evolved since its introduction. It offers a noninvasive method of assessing tissue stiffness, providing crucial insights into various medical conditions [54,57,58,59]. Primarily used in endosonography, elastography is invaluable for diagnosing gastrointestinal ailments, particularly pancreatic diseases such as cancer, pancreatitis, and autoimmune pancreatitis [57]. It aids in differentiating between malignant and benign lesions, which is critical for effective disease management and treatment planning.

There are two primary forms of elastography utilized in clinical settings: strain elastography and shear wave elastography [57,58,59]. Strain elastography visualizes tissue stiffness using a color scale, while shear wave elastography offers quantitative data on tissue rigidity, enhancing diagnostic accuracy [57]. These methods have been integrated with other advanced techniques, such as contrast-enhanced endoscopic ultrasound (EUS), to further refine diagnostic capabilities. When combined, these techniques significantly improve the identification of pancreatic lesions, providing high sensitivity and specificity in detecting pancreatic cancer [57,58].

However, despite its benefits, elastography faces challenges such as the need for standardized procedures and clear diagnostic thresholds [54,57,59]. Ongoing research and development are essential to overcome these limitations and fully leverage elastography’s potential in clinical practice. Future studies should focus on enhancing the methodology, standardizing application protocols, and expanding its use in other medical fields such as liver fibrosis, breast cancer, and prostate cancer, where it has already shown promising results [60,61,62].

This integrated approach to pancreatic imaging, combining elastography with other diagnostic modalities, represents a significant advancement in the field. It not only improves the accuracy of diagnoses but also facilitates early detection and efficient management of pancreatic disorders, paving the way for more personalized and targeted treatment strategies.

### Present Application, Challenges, and Prospects of Elastography

Combining contrast-enhanced endoscopic ultrasound (EUS) with EUS elastography enhances pancreatic cancer diagnostics by improving vascular visualization within the pancreas and assessing tissue elasticity. This synergy aids in distinguishing between malignant and benign lesions, offering crucial insights for early detection and management. Elastography, specifically, has proven effective in differentiating pancreatic lesions, which can influence treatment strategies and patient monitoring. Despite its promise, the approach faces challenges such as standardization and establishing clear diagnostic thresholds. Elastography (both the strain and shear wave forms) provides valuable noninvasive insights into tissue stiffness, extending its potential beyond pancreatic applications to liver fibrosis and various cancers. Strain elastography visually maps tissue stiffness, while shear wave elastography quantitatively measures it, enhancing diagnostic precision. Yet, the widespread application of these techniques is limited by the need for further standardization and research to optimize protocols and ensure reliable interpretations of elastographic data. In summary, while elastography’s integration into clinical practice holds the potential to significantly improve pancreatic disease diagnostics, ongoing efforts are crucial to address existing challenges and fully harness its capabilities. Future advancements in elastography promise to refine the diagnostic process across a range of medical conditions, necessitating continued interdisciplinary collaboration to achieve standardized, optimized imaging techniques for enhanced patient care.

## 9. Contemporary Pancreatic Imaging Modalities-Limitations and the Future

Endoscopic ultrasound (EUS) and its elastography enhancement offer significant diagnostic advantages, particularly in pancreaticobiliary diseases [62]. By evaluating tissue elasticity, EUS elastography improves the distinction between malignant and benign tissues, and by identifying hard and soft tissues in real-time, the precision of fine-needle aspiration (FNA) facilitates prompt decision-making for patient management. Despite its benefits, EUS elastography faces challenges, such as difficulties in controlling tissue compression and obtaining clear images near rigid structures or in areas with scant soft tissue. Advancements in software have mitigated some of these limitations, and its use is expanding, particularly in centers specializing in pancreatic diseases, without necessitating additional training for adept EUS endoscopists. Future applications of elastography might include assessing residual cancer post-radiotherapy or diagnosing vessel infiltration by pancreatic cancer, although these are currently under exploration [63]. The technique encompasses qualitative and quantitative methods; qualitative (strain elastography) uses a color scale for tissue stiffness, while quantitative methods, such as EUS shear wave measurement (SWM), offer numerical stiffness values, allowing for more nuanced assessments across different conditions or individuals [64]. Elastography’s potential for improving diagnostic accuracy for pancreatic lesions underscores the need for its integration into clinical practice, promising enhanced diagnostic confidence and better treatment planning.

Some diseases, however, especially pancreatic pathologies, are trickier to diagnose due to their presentation. In the diagnostic domain of pancreatic diseases, particularly when distinguishing autoimmune pancreatitis (AIP) from pancreatic ductal adenocarcinoma (PDAC), the limitations of imagistic methods necessitate alternative diagnostic approaches. Imaging techniques, while essential, often fall short due to the overlapping radiological features exhibited by AIP and PDAC, which can lead to diagnostic ambiguity. This limitation underscores the importance of serological tests, which offer a more definitive diagnostic utility. Advanced serological markers such as IgG glycosylation patterns have shown promise in distinguishing these conditions with high accuracy. Studies have demonstrated that specific glycan biomarkers can significantly refine the diagnostic process, providing a clear distinction between AIP and PDAC that imaging alone might not achieve [65]. Additionally, combining serum markers such as CA 19-9 with globulin levels and eosinophil percentages has been effectively utilized to establish diagnostic thresholds, which is critical given the similar imaging profiles of these diseases [66,67,68]. Therefore, serological testing emerges as a crucial diagnostic tool, especially when trying to diagnose diseases such as autoimmune pancreatitis, complementing imaging methods and guiding appropriate therapeutic interventions.

## 10. Results

The assembled and gathered data shows the efficacy of various techniques in discerning the nature and extent of neoplastic lesions within the pancreas. Among the imaging modalities assessed, multidetector computed tomography (MDCT) and magnetic resonance imaging (MRI) are paramount. MDCT exhibits a commendable diagnostic accuracy of 83.3%, while MRI surpasses this with an accuracy of 89.1%. These modalities, pivotal in staging pancreatic carcinoma, are further complemented by the precision of endoscopic ultrasound (EUS), which initially demonstrated an 82% accuracy rate. Notably, this accuracy surged to 93.7% with the incorporation of contrast enhancement and elastography, which are particularly efficacious for tumors less than 20 mm in diameter, underscoring EUS’s superior resolution in such cases.

Emerging noninvasive techniques such as elastography have been identified as particularly promising. This method, which maps the elastic properties and stiffness of soft tissue, provides critical diagnostic insights which are especially useful in the early detection of pathologies such as fibrosis and neoplasms. Additionally, fine needle aspiration (FNA), a minimally invasive procedure, offers a rapid and effective means of obtaining tissue samples for histopathological examination, with results typically available within a concise timeframe. The procedural synergy of FNA with imaging modalities enhances the diagnostic landscape, allowing for nuanced differentiation between benign and malignant pancreatic entities.

The importance of adopting a multimodal imaging approach is clear, combining the anatomical and functional insights provided by CT, MRI, and EUS. This integrative strategy not only delineates the pancreatic architecture and associated anomalies with enhanced clarity but also facilitates targeted therapeutic interventions based on precise pathological assessments. Moreover, advancements such as quantitative shear wave elastography (SWE) enrich the diagnostic toolkit by quantifying tissue stiffness, thereby bolstering the accuracy in distinguishing malignancies, which is pivotal for formulating optimal management strategies for pancreatic tumors. These findings collectively underscore the transformative potential of sophisticated imaging techniques in the realm of pancreatic oncology, heralding a new era of precision medicine.

## 11. Conclusions

The most common imaging modalities currently used in diagnosing pancreatic neoplasms are multidetector computed tomography (MDCT), endoscopic ultrasound (EUS), and magnetic resonance imaging (MRI). These technologies are integral in distinguishing between benign and malignant pancreatic lesions, offering a high level of diagnostic accuracy that is crucial for optimal therapeutic planning. However, each of them has strengths and weaknesses; some are better in combinations with others.

MDCT is renowned for its detailed visualization capabilities, providing essential data on the staging of pancreatic carcinoma through rapid acquisition and high-resolution images. These features are indispensable for assessing tumor size, location, and involvement with adjacent structures, which are vital for staging and determining treatment pathways.

EUS, particularly when combined with fine needle aspiration (FNA), is invaluable for directly visualizing pancreatic ducts and nearby tissues. This technique supports precise lesion localization and facilitates tissue sample collection for cytological analysis, aiding in differential diagnosis among inflammatory, benign, and malignant pathologies.

Enhancing these techniques with contrast agents and EUS elastography significantly augments the ability to define the vascular architecture within lesions, a common indicator of malignancy due to the increased vascular activity associated with cancerous growths. This integration markedly boosts diagnostic accuracy for pancreatic cancer, underscoring the critical role of advanced imaging in the early detection and management of this disease.

Despite these advantages, several challenges hinder their universal application, including the lack of standardized protocols and the high level of expertise required for image interpretation. These factors can limit their use to specialized centers, affecting general accessibility in clinical practice.

Anticipated advances in imaging technology, alongside efforts toward protocol standardization and professional training, are expected to address these limitations. Future improvements may include enhanced image resolution, sophisticated processing algorithms, and more intuitive user interfaces, which could democratize the use of these advanced techniques across a broader range of healthcare settings. The development of artificial intelligence in medical imaging also holds the potential to support clinicians by providing automated, accurate image analysis tools.

When considering which methods are best based on accuracy, MRI is often considered superior due to its exceptional soft tissue contrast and ability to produce high-quality images without ionizing radiation, making it particularly useful for the detailed characterization of pancreatic lesions. MDCT, while slightly less accurate in soft tissue contrast, provides quicker scans and is more widely available, making it better suited for initial assessments and emergency situations. EUS, when combined with FNA, offers the highest specificity and sensitivity for diagnosing pancreatic cancer, particularly when evaluating lesions that are not clearly delineated by other imaging methods. The combination of EUS with contrast-enhanced techniques and elastography often provides the most comprehensive data, allowing clinicians to make the most informed decisions regarding the nature of pancreatic lesions and the best course of treatment.

Therefore, while each imaging method has its strengths, the combination of EUS with advanced techniques often yields the highest diagnostic accuracy, making it the preferred choice in clinical settings where available, particularly for complex cases requiring the detailed analysis of pancreatic tissue.

## Figures and Tables

**Table 1:** Common pancreatic cysts and characteristics.

Type of Cyst	Benign/Malignant	Common Imaging Methods	Characteristics	Management Notes
Serous cystadenoma	Benign	CT, MRI, EUS	Microcystic appearance, central scar, usually asymptomatic	Regular monitoring; surgery if symptomatic or increased growth
Mucinous cystadenoma	Pre-malignant	MRI, EUS	Macrocytic appearance, septations, mucin-producing	Surgical resection in case of potential of malignancy
Intraductal papillary mucinous neoplasm (IPMN)	Benign to malignant	MRI, MRCP, EUS	Dilation of pancreatic ducts, mural nodules	Surveillance; resection if high-risk stigmata present
Mucinous cystic neoplasm (MCN)	Pre-malignant to malignant	CT, MRI, EUS	Ovarian-type stroma, typically in the body/tail of pancreas	Surgical resection advised in some cases due to malignancy risk
Solid pseudopapillary neoplasm	Low-grade malignant	CT, MRI	Solid and cystic components, hemorrhagic content	Surgical resection due to potential for metastasis
Pseudocyst	Benign	CT, EUS	Associated with pancreatitis, lack of epithelial lining	Endoscopic or surgical drainage if symptomatic

**Table 2:** M.I. Costache and Christoph F. Dietrich’s studies on imaging of pancreatic masses.

Pathology	Method of Examination	Specificity	Sensibility	Reference
Pancreatic cancer	Contrast enhancement and elastography	90.3%	97.5%	Which is the Best Imaging Method in Pancreatic Adenocarcinoma Diagnosis and Staging- CT, MRI or EUS?—M.I. Costache et al. [3]
Pancreatic cancer	Quantitative EUS elastography	16.7% and 22.2%	100% and 95.7%	Elastography of the Pancreas, Current View—Christoph F. Dietrich [40]

## Data Availability

Not applicable.

## Abbreviations

MDCT	Multidetector computed tomography
MRI	Magnetic resonance imaging
MRCP	Magnetic resonance cholangiopancreatography
CT	Computed tomography
EUS	Endoscopic ultrasound
US	Ultrasound
SI	Strain index
BMI	Body mass index
SWE	Shear wave elastography
SWV	Shear wave velocity
AUC	Under the curve
ROC	Operating characteristic
PLC	Pancreatic lesion cyst
PCL	Pancreatic cystic lesions
PCN	Pancreatic cystic neoplasm
FNAC	Fine needle aspiration cytology
FNA	Fine needle aspiration
RTE	Real-time tissue elastography
EUS-FNA	Endoscopic ultrasound-guided fine needle aspiration
SWM	Shear wave measurement
ESMO	The European Society of Medical Oncology
